# Arrhythmogenic Left Ventricular Cardiomyopathy Associated with *MYH7* Mutation

**DOI:** 10.19102/icrm.2026.17104

**Published:** 2026-10-15

**Authors:** Afnan Chaudhry, Ibrahim Halil Inanc, Celebi Yildirim, Daniel Ratliff, Bidhya Timilsina

**Affiliations:** 1 Phoenixville Hospital Tower Health, Phoenixville, PA, USA

**Keywords:** Arrhythmogenic cardiomyopathy, cardiac magnetic resonance, late gadolinium enhancement, left-dominant cardiomyopathy, *MYH7*

## Abstract

Arrhythmogenic left ventricular cardiomyopathy (ALVC) is an increasingly recognized phenotype within the spectrum of arrhythmogenic cardiomyopathies, historically associated with mutations in desmosomal genes. However, emerging evidence suggests that non-desmosomal variants may also contribute to this disease entity. We report the case of a 58-year-old man with atrial fibrillation, nonischemic cardiomyopathy, and prior cryptogenic stroke who presented with progressive exertional dyspnea, chest discomfort, and palpitations. Coronary angiography excluded obstructive coronary artery disease. Transthoracic echocardiography demonstrated reduced left ventricular systolic function with features suggestive of infiltrative cardiomyopathy. A technetium-99m pyrophosphate scan was negative for transthyretin amyloidosis. Cardiac magnetic resonance imaging ultimately revealed extensive mid-wall late gadolinium enhancement involving multiple basal and mid-left ventricular segments in a nonischemic distribution, consistent with myocardial fibrosis. Comprehensive genetic testing identified a heterozygous pathogenic *MYH7* variant (p.Arg723Cys), fulfilling the genetic component of the Padua criteria for ALVC. Rhythm control was achieved with electrical cardioversion and maintained with dofetilide, while heart failure was managed with guideline-directed medical therapy. In view of the extensive myocardial fibrosis and reduced left ventricular ejection fraction, primary prevention with an implantable cardioverter-defibrillator was recommended. This case expands the phenotypic spectrum of *MYH7*-associated cardiomyopathy and highlights the critical role of multimodal imaging and genetic testing in establishing the diagnosis of ALVC and guiding risk stratification.

**CLINICAL IMPACT:**

Arrhythmogenic left ventricular cardiomyopathy (ALVC) remains an underrecognized and diagnostically challenging phenotype of arrhythmogenic cardiomyopathy. This case describes a potential association between the pathogenic MYH7 p.Arg723Cys variant and ALVC, expanding the phenotypic spectrum of MYH7-associated cardiomyopathy beyond its more established associations with hypertrophic and dilated cardiomyopathy. The case highlights the complementary roles of cardiac magnetic resonance imaging and genetic testing in identifying ALVC when conventional evaluation is inconclusive. Recognition of extensive myocardial fibrosis together with a pathogenic genetic variant may also provide important information for arrhythmic risk stratification, consideration of primary-prevention implantable cardioverter-defibrillator therapy, genetic counseling, and cascade testing of first-degree relatives.

## Introduction

Arrhythmogenic cardiomyopathy (ACM) is an inherited heart muscle disorder affecting about 1 in 5000 people, most often associated with mutations in desmosomal protein genes responsible for cardiomyocyte coupling.^1^ ACM is characterized by fibrofatty or fibrous myocardial infiltration, leading to impaired systolic ventricular function, ventricular arrhythmias, and an increased risk of sudden cardiac death. Traditionally viewed as a right ventricular disease, advances in imaging have uncovered variants with biventricular and isolated left ventricular (LV) involvement, leading to the classification of arrhythmogenic LV cardiomyopathy (ALVC).^2^ ALVC can present similarly to other genetic and non-genetic conditions such as dilated cardiomyopathy, cardiac sarcoidosis, myocarditis, and neuromuscular cardiomyopathies. Comprehensive clinical assessment, family history, and multimodal imaging are essential for differentiation, but a definitive diagnosis of ALVC ultimately depends on genetic confirmation of a pathogenic variant in an ACM-related gene.^1^ Our case demonstrates an association between the *MYH7* p.Arg723Cys missense mutation and ALVC.

## Case presentation

A 58-year-old man with a history of atrial fibrillation anticoagulated with rivaroxaban, nonischemic cardiomyopathy, and prior cryptogenic stroke was evaluated for progressive exertional dyspnea, chest discomfort, and palpitations. Initial investigations revealed atrial fibrillation/flutter with variable block, an LV ejection fraction (LVEF) of 41%, and echocardiographic findings suggestive of infiltrative cardiomyopathy **(Figure 1)**. A technetium-99m pyrophosphate scan was negative, effectively ruling out transthyretin amyloidosis.

**Figure 1:**
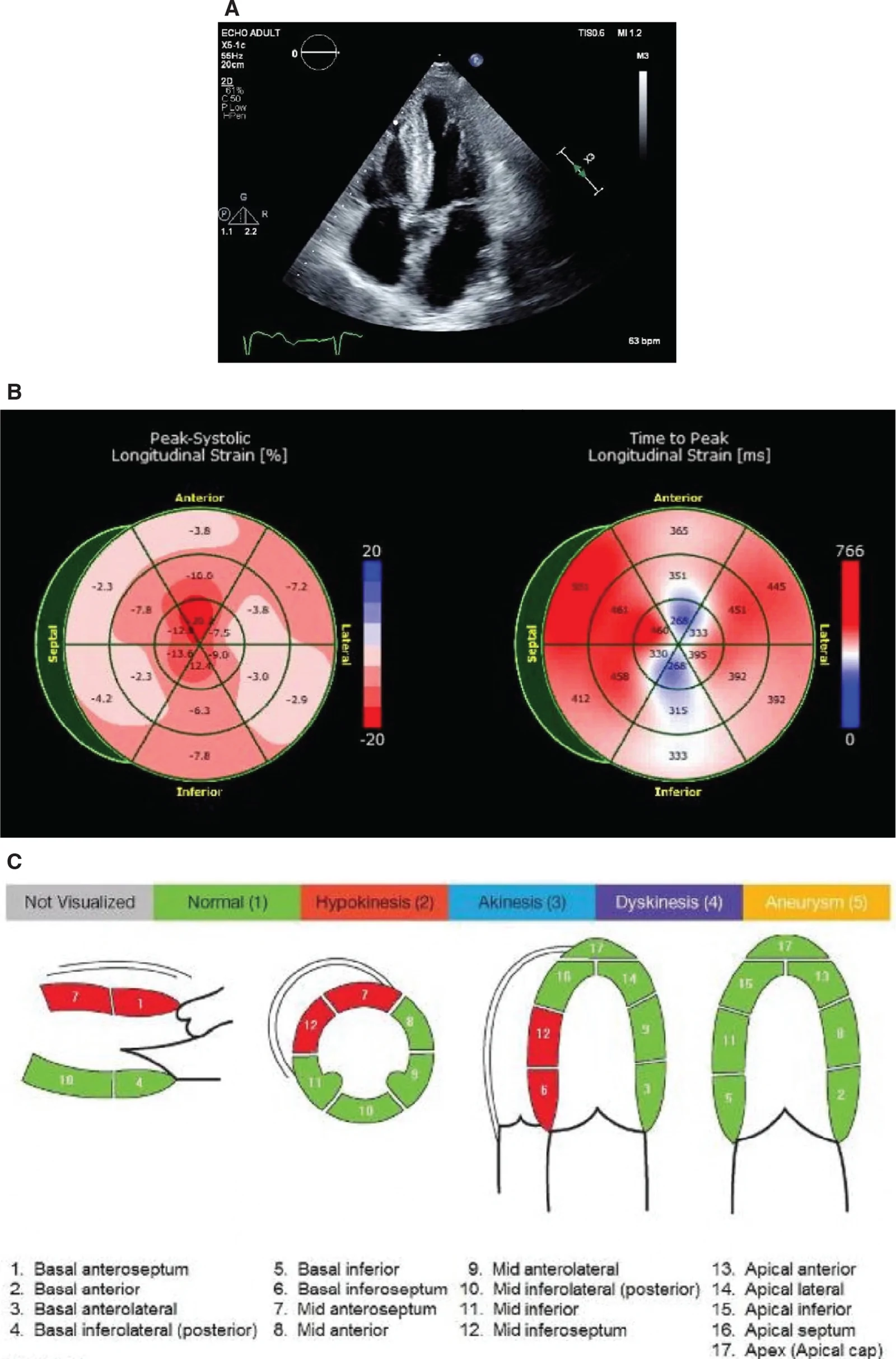
Transthoracic echocardiographic findings. **A:** Apical four-chamber transthoracic view demonstrating normal left ventricular cavity size with concentric hypertrophy, trabeculation, and speckled myocardial appearance. **B:** Peak systolic longitudinal strain bull’s-eye map showing markedly reduced global longitudinal strain (−7.8%) with diffuse impairment, although the apex is relatively less affected. **C:** Segmental wall motion analysis demonstrating relative hypokinesis of the basal-to-mid anteroseptal and inferoseptal segments.

Cardiac magnetic resonance imaging (CMR) was then performed and revealed mildly reduced systolic function; a nondilated right ventricle; and extensive mid-wall late gadolinium enhancement (LGE) affecting multiple basal and mid-LV segments, including the anterior septum, inferior septum, and anterior, inferior, and inferolateral wall segments **(Figure 2)**. The pattern of fibrosis was nonischemic, without corresponding coronary artery disease on angiography, and was consistent with a diagnosis of ALVC.

**Figure 2:**
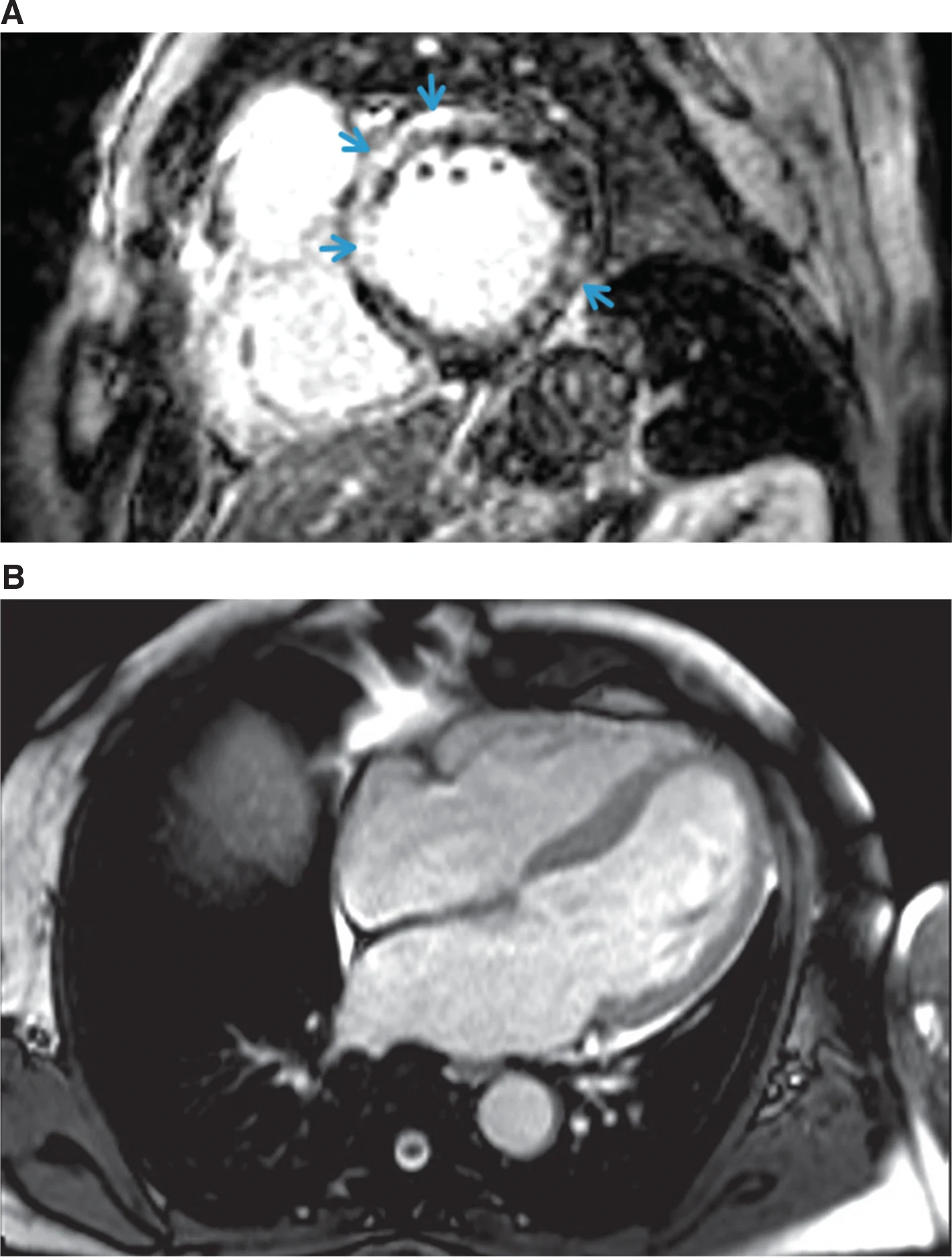
Cardiac magnetic resonance imaging. **A:** Late gadolinium enhancement sequence demonstrating extensive, preferentially mid-wall fibrosis involving the basal and mid-left ventricular segments in a nonischemic pattern. **B:** Four-chamber view showing a mildly to moderately dilated left ventricle.

Comprehensive genetic testing identified a heterozygous pathogenic variant in the *MYH7* gene (c.2167C>T, p.Arg723Cys), a missense mutation previously implicated in hypertrophic, dilated, and arrhythmogenic cardiomyopathies. Additional variants of uncertain significance were noted in *KCNA1* and *MYOM1*. The *MYH7*-related cardiomyopathy is inherited in an autosomal dominant fashion and is known to manifest with overlapping phenotypes, including LV noncompaction and arrhythmia susceptibility.

Sinus rhythm was restored through electrical cardioversion and maintained with dofetilide **(Figure 3)**. Heart failure with reduced ejection fraction was managed using guideline-directed medical therapy, including metoprolol succinate, dapagliflozin, sacubitril–valsartan, and eplerenone. Given the presence of a pathogenic *MYH7* mutation, extensive mid-wall fibrosis on CMR, and an LVEF of <45%, the patient was deemed to be at elevated risk for malignant ventricular arrhythmias and sudden cardiac death. β-Blocker therapy with metoprolol was continued, and primary prevention with a prophylactic implantable cardioverter-defibrillator (ICD) was recommended. Genetic counseling was provided, and cascade testing was advised for first-degree relatives.

**Figure 3:**
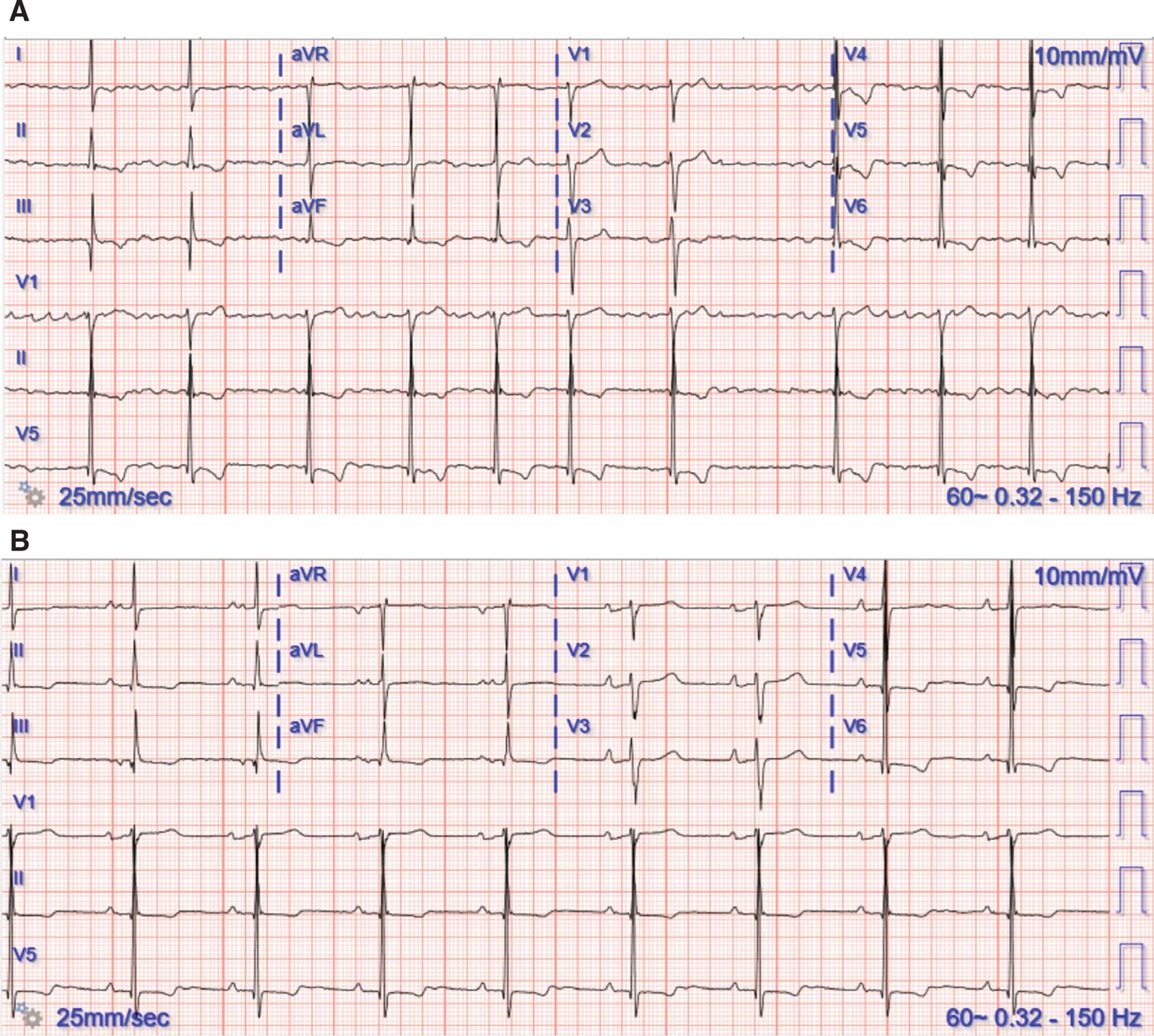
Twelve-lead electrocardiograms (ECGs). **A:** Presenting ECG demonstrating atrial fibrillation/flutter with variable block, minimal voltage criteria for left ventricular hypertrophy, and lateral ST–T-wave abnormalities. **B:** Post-cardioversion ECG showing restoration of sinus rhythm with sinus bradycardia, first-degree atrioventricular block, and left ventricular hypertrophy with repolarization abnormality.

Institutional review board approval was not required for this study, as it represents a single-patient case report with no prospective intervention, no research-related procedures, and no identifiable patient information.

## Discussion

Genetic findings are a key part of ACM diagnosis. However, ACM is genetically diverse, with mutations inherited in dominant or recessive patterns and influenced by factors such as age, sex, and physical activity. Some mutations lack a clear genotype–phenotype correlation and are classified as variants of uncertain significance, while others remain gene-elusive despite evident familial inheritance; some are even present in the normal population as “genetic noise.” These limitations challenge accurate diagnosis and genetic counseling. Advancing our understanding of ACM genetics is critical for improving risk stratification and developing targeted therapies.^2^

The most frequently involved genes in ACM are those encoding desmosomal proteins, such as plakophilin-2, desmoglein, desmoplakin, desmocollin, and plakoglobin (JUP).^3^ The *MYH7* gene encodes the β-myosin heavy chain, a key structural protein in cardiac and skeletal muscle fibers. Mutations in *MYH7* have been associated with a spectrum of cardiomyopathies, most commonly with over 200 variants linked to dilated and hypertrophic cardiomyopathy and less frequently with approximately 30 variants implicated in LV noncompaction.^4^ Two variants, c.2630T>C and c.2609G>A, have previously been reported as causes of ACM.^5^ Our case highlights a potential association between the p.Arg723Cys variant and ALVC. While there is ongoing research to correlate specific genotypes with clinical phenotypes, current data on individual mutations remain too limited to provide definitive clinical guidance.^4^

The diagnostic framework for ACM has advanced with the introduction of the 2020 Padua criteria, which enhances accuracy by incorporating CMR alongside electrocardiogram and echocardiographic findings. CMR enables noninvasive detection of LGE and myocardial fibrosis, which are hallmarks of ACM. ALVC is diagnosed when a major structural abnormality is present alongside a pathogenic ACM mutation. Arrhythmogenic right ventricular cardiomyopathy (ARVC) is classified as definite with either two major criteria, one major plus two minor, or four minor criteria from separate categories; borderline with either one major and one minor or three minor criteria; and possible with either one major or two minor criteria. Biventricular ACM is diagnosed when diagnostic criteria are met for both right and LV involvement.^6^

The Padua criteria for diagnosing ALVC are divided into six categories. Morphofunctional abnormalities include minor criteria such as global LV systolic dysfunction and regional wall motion abnormalities such as hypokinesia or akinesia. Structural abnormalities feature a major criterion: the presence of LGE in the LV free wall or septum. Repolarization abnormalities are defined by minor criteria such as T-wave inversions in leads V4–V6 in the absence of complete left bundle branch block. Depolarization abnormalities include low QRS voltages (<0.5 mV peak to peak) in the limb leads, excluding secondary causes such as obesity or emphysema. Ventricular arrhythmias are considered minor and include frequent ectopic beats (>500 per 24 h) or non-sustained or sustained ventricular tachycardia with a right bundle branch block morphology, excluding fascicular patterns. Family history and genetics contribute a major criterion if there is a confirmed ACM diagnosis in a first-degree relative or identification of a pathogenic mutation in the patient; minor criteria include a suggestive but unconfirmed family history or premature sudden death under age 35 years in a close relative.^6^

There are no specific management recommendations for ALVC or biventricular ACM, although they are now established as distinct diagnoses. General treatment strategies for ACM focus on controlling ventricular arrhythmia with β-blocker therapy and treating heart failure with guideline-directed medical therapy. The effectiveness of other anti-arrhythmics, such as flecainide, remains less certain. In patients with frequent ventricular tachycardia or appropriate ICD shocks despite β-blocker use, catheter ablation is preferred. ICD implantation is indicated for all patients for secondary prevention and for those with an LVEF of <35%. For primary prevention, there is no universal consensus, although ICDs are generally favored in patients at high risk, ie, those with an estimated annual risk of life-threatening arrhythmias of >10% based on factors including arrhythmic burden, systolic dysfunction, history of syncope, sex, genotype, and endocardial voltage mapping findings. High-risk phenotypes, such as ALVC and biventricular ACM, also support consideration for ICD placement. However, a formal risk calculator has only been developed for ARVC by the European Society of Cardiology. Genetic testing is recommended for all first-degree relatives. Relatives who test negative for the familial variant can be discharged from further follow-up, while those who test positive should be enrolled in long-term monitoring.^7^ As CRISPR technology advances, a deeper understanding of genetic mutations and their underlying mechanisms may enable the development of personalized therapies.^8^

## Conclusion

This case highlights a potential association between the *MYH7* p.Arg723Cys mutation and ALVC, expanding the genotypic spectrum beyond traditionally implicated desmosomal genes. ALVC remains a diagnostic and clinical challenge due to the complexity of genetic interpretation. The 2020 Padua criteria have improved diagnostic accuracy through the incorporation of advanced CMR and genetic findings. However, management strategies remain extrapolated from broader ACM guidelines, with particular uncertainty surrounding risk stratification and primary prevention in ALVC. As our understanding of genotype–phenotype correlations continues to evolve, further research is essential to clarify the pathogenicity of non-desmosomal variants, refine risk prediction, and develop targeted therapies.
